# Supplementary material for: Tumor Cell-Associated IL-1α Affects Breast Cancer Progression and Metastasis in Mice through Manipulation of the Tumor Immune Microenvironment
Source: Int J Mol Sci. 2024 Apr 2;25(7):3950. doi: 10.3390/ijms25073950 (PMC11011794; doi:10.3390/ijms25073950)
Supplement: Supplementary file 1 [file ijms-25-03950-s001.zip › Supplementary tables.pdf]

Supplementary Table S1. List of genes within the enriched KEGG pathways (Fig. 2B) upregulated in IL-1α KO cells.

gene	GENENAME	linearFC	Padj
Angpt1	angiopoietin 1	10.57	0.042713826
Arap3	ArfGAP with RhoGAP domain ankyrin repeat and PH domain 3	24.13	5.12366E-47
Bcl2l1	BCL2-like 1	2.27	4.78571E-05
Bcl3	B cell leukemia/lymphoma 3	9.44	1.68901E-10
Bdnf	brain derived neurotrophic factor	5.52	0.030694487
Cacna1e	calcium channel voltage-dependent R type alpha 1E subunit	10.58	0.037531082
Ccnd1	cyclin D1	2.49	0.01346269
Cd14	CD14 antigen	4.08	3.40571E-14
Cdk6	cyclin-dependent kinase 6	2.36	2.01364E-06
Csf3	colony stimulating factor 3 (granulocyte)	3.48	0.000620474
Cyp1b1	cytochrome P450 family 1 subfamily b polypeptide 1	6.97	3.15852E-19
Cyp24a1	cytochrome P450 family 24 subfamily a polypeptide 1	7.81	9.01993E-05
Dab2ip	disabled 2 interacting protein	2.12	0.000352617
Dll4	delta like canonical Notch ligand 4	3.08	6.17365E-06
Dusp6	dual specificity phosphatase 6	3.25	3.61807E-15
Ednra	endothelin receptor type A	18.83	3.17426E-06
Egln3	egl-9 family hypoxia-inducible factor 3	2.20	0.037305193
Etv1	ets variant 1	2.13	0.007033183
Etv4	ets variant 4	1.96	0.04144882
Fgfr1	fibroblast growth factor receptor 1	6.93	0.004647439
Fgfr3	fibroblast growth factor receptor 3	3.02	0.020848078
Fos	FBJ osteosarcoma oncogene	11.24	0.006994652
Gnao1	guanine nucleotide binding protein alpha O	5.78	1.3338E-08
Gngt2	guanine nucleotide binding protein (G protein) gamma transducing activity polypeptide 2	9.92	0.031251511
Gstm2	glutathione S-transferase mu 2	4.20	0.00028536

Hhip	Hedgehog-interacting protein	2.93	3.69114E-08
Hspb1	heat shock protein 1	15.05	0.047667228
Il1r1	interleukin 1 receptor type I	2.34	0.040535609
Il27ra	interleukin 27 receptor alpha	8.08	0.000241155
Il33	interleukin 33	11.70	5.38164E-07
Il6st	interleukin 6 signal transducer	2.07	0.000464181
Itga1	integrin alpha 1	9.36	2.59492E-06
Itga7	integrin alpha 7	4.85	0.007670691
Itga8	integrin alpha 8	6.14	0.012319146
Itgb2	integrin beta 2	5.47	5.85471E-15
Itgb7	integrin beta 7	4.57	0.005582357
Jak2	Janus kinase 2	2.84	0.001110239
Lbp	lipopolysaccharide binding protein	22.00	1.45316E-08
Lcn2	lipocalin 2	5.28	8.456E-05
Map3k6	mitogen-activated protein kinase kinase kinase 6	3.37	0.000118428
Mgst2	microsomal glutathione S-transferase 2	3.47	0.005052301
Myd88	myeloid differentiation primary response gene 88	2.89	1.95538E-08
Nckap1l	NCK associated protein 1 like	24.51	1.08593E- 05
Nfe2l2	nuclear factor erythroid derived 2 like 2	2.77	1.44164E-14
Notch1	notch 1	2.40	0.004257293
Nqo1	NAD(P)H dehydrogenase quinone 1	8.77	1.84507E-12
Nr4a1	nuclear receptor subfamily 4 group A member 1	2.78	0.031733882
Pcna	proliferating cell nuclear antigen	2.19	0.037305193
Pdgfa	platelet derived growth factor alpha	1.57	0.002912728
Pdgfra	platelet derived growth factor receptor alpha polypeptide	3.06	0.010347306
Pgf	placental growth factor	2.80	0.003703585
Pla1a	phospholipase A1 member A	4.63	2.91141E-09
Plaur	plasminogen activator urokinase receptor	2.28	0.004866973
Prex1	phosphatidylinositol-3 4 5-trisphosphate-dependent Rac exchange factor 1	6.14	1.72031E-05

Prkch	protein kinase C eta	10.08	1.668E-36
Ptgs2	prostaglandin-endoperoxide synthase 2	2.87	0.031293977
Rap1b	RAS related protein 1b	1.93	0.006265006
Rapgef3	Rap guanine nucleotide exchange factor (GEF) 3	2.15	0.030202385
Sdc4	syndecan 4	2.32	0.002723489
Selp	selectin platelet	20.53	1.07648E-30
Sema3b	sema domain immunoglobulin domain (Ig) short basic domain secreted (semaphorin) 3B	3.58	3.20518E-05
Sema6d	sema domain transmembrane domain (TM) and cytoplasmic domain (semaphorin) 6D	4.86	0.00010719
Serpinf1	serine (or cysteine) peptidase inhibitor clade F member 1	9.04	0.002001252
Sfrp1	secreted frizzled-related protein 1	3.56	0.009689426
Sgk1	serum/glucocorticoid regulated kinase 1	3.54	3.03936E-18
Shc1	src homology 2 domain-containing transforming protein C1	2.31	0.0039014
Sipa1l2	signal-induced proliferation-associated 1 like 2	2.28	0.020848078
Socs2	suppressor of cytokine signaling 2	3.25	4.93412E-05
Socs3	suppressor of cytokine signaling 3	3.37	0.000285488
Spn	sialophorin	2.36	0.011138712
Src	Rous sarcoma oncogene	2.41	1.36126E-06
Srgap3	SLIT-ROBO Rho GTPase activating protein 3	2.61	0.013908215
Sv2a	synaptic vesicle glycoprotein 2 a	3.88	0.02508398
Tgfb1	transforming growth factor beta 1	2.02	7.02472E-16
Tlx3	T cell leukemia homeobox 3	4.30	0.031251511
Tnfrsf19	tumor necrosis factor receptor superfamily member 19	15.81	1.69274E-28
Traf5	TNF receptor-associated factor 5	3.49	0.000433313
Trim71	tripartite motif-containing 71	8.30	0.008512807
Tslp	thymic stromal lymphopoietin	4.23	6.38265E-10
Twist1	twist basic helix-loop-helix transcription factor 1	1.95	2.41724E-12
Vegfa	vascular endothelial growth factor A	1.78	0.018043413

Vegfc	vascular endothelial growth factor C	2.85	0.001122909
Vim	vimentin	1.84	2.51953E-12
Ywhab	tyrosine 3-monooxygenase/tryptophan 5-monooxygenase activation protein beta polypeptide	2.07	1.45505E-05
Zeb2	zinc finger E-box binding homeobox 2	4.26	1.38197E-11

Supplementary Table S2. List of genes within the enriched KEGG pathways (Fig. 2B) downregulated in IL-1*α* KO cells.

gene	GENENAME	linearFC	padj
Ablim1	actin-binding LIM protein 1	-4.34	3.6481E-09
Alcam	activated leukocyte cell adhesion molecule	-10.41	7.3872E-17
Ank3	ankyrin 3 epithelial	-6.83	2.8269E-07
Blnk	B cell linker	-40.25	3.4959E-07
Bmp7	bone morphogenetic protein 7	-15.59	0.00109181
Camk2b	calcium/calmodulin-dependent protein kinase II beta	-5.34	0.00202162
Card14	caspase recruitment domain family member 14	-14.34	0.00136385
Cav1	caveolin 1 caveolae protein	-3.36	2.9007E-05
Ccl2	chemokine (C-C motif) ligand 2	-5.88	2.8587E-06
Ccl20	chemokine (C-C motif) ligand 20	-3.24	0.00825271
Ccn4	cellular communication network factor 4	-4.04	1.4856E-06
Cdh1	cadherin 1	-49.09	5.835E-109
Cldn23	claudin 23	-5.52	0.00068005
Cldn4	claudin 4	-103.94	2.1711E-54
Cldn7	claudin 7	-13.56	4.6068E-16
Cldn9	claudin 9	-32.27	4.0511E-14
Cntfr	ciliary neurotrophic factor receptor	-15.82	0.00089444
Crb3	crumbs family member 3	-25.42	1.6105E-10
Csf1r	colony stimulating factor 1 receptor	-21.92	0.00026901
Csf2	colony stimulating factor 2 (granulocyte-macrophage)	-31.41	7.2622E-06
Dapk2	death-associated protein kinase 2	-7.71	0.01175151

Ddit4	DNA-damage-inducible transcript 4	-3.20	5.6917E-07
Dvl1	dishevelled segment polarity protein 1	-2.38	0.00582442
Efna3	ephrin A3	-5.35	0.00199318
Efnb1	ephrin B1	-14.38	3.0737E-33
Epb41l4b	erythrocyte membrane protein band 4.1 like 4b	-15.58	6.0272E-15
Epcam	epithelial cell adhesion molecule	-104.71	7.8035E-89
Epha8	Eph receptor A8	-19.93	4.9854E-05
Ephb3	Eph receptor B3	-11.29	8.6312E-13
Ephb6	Eph receptor B6	-56.72	9.5267E-11
Erbb3	erb-b2 receptor tyrosine kinase 3	-16.11	8.0835E-08
Ets2	E26 avian leukemia oncogene 2 3' domain	-4.75	2.8561E-19
Ezh2	enhancer of zeste 2 polycomb repressive complex 2 subunit	-2.17	0.00211688
Fas	Fas (TNF receptor superfamily member 6)	-5.65	0.00196027
Fn1	fibronectin 1	-3.43	3.6239E-06
Gadd45b	growth arrest and DNA-damage-inducible 45 beta	-19.55	1.0253E-12
Gls2	glutaminase 2 (liver mitochondrial)	-2.93	0.00020406
Gng11	guanine nucleotide binding protein (G protein) gamma 11	-3.55	2.2471E-07
Gng7	guanine nucleotide binding protein (G protein) gamma 7	-12.98	0.00213662
Gstm5	glutathione S-transferase mu 5	-3.75	0.01673483
Gsto1	glutathione S-transferase omega 1	-2.73	5.7814E-12
Gstt1	glutathione S-transferase theta 1	-55.23	1.4586E-08
Ifngr1	interferon gamma receptor 1	-2.18	0.02099747
Ifnlr1	interferon lambda receptor 1	-66.49	2.7449E-12
Il15ra	interleukin 15 receptor alpha chain	-4.68	0.00571329
Il17re	interleukin 17 receptor E	-59.62	4.0052E-09
Il18	interleukin 18	-16.04	0.0008664
Il18rap	interleukin 18 receptor accessory protein	-59.09	2.4977E-11
Il1b	interleukin 1 beta	-10.37	0.02729636
Il23a	interleukin 23 alpha subunit p19	-5.19	4.4866E-09

Il24	interleukin 24	-91.02	4.6138E-25
Inhba	inhibin beta-A	-15.89	1.0065E-45
Irf1	interferon regulatory factor 1	-3.48	0.00133808
Itga2	integrin alpha 2	-3.58	0.04365866
Itgb4	integrin beta 4	-6.34	1.1509E-42
Itgb5	integrin beta 5	-2.88	0.00192506
Itgb6	integrin beta 6	-41.50	1.3818E-08
Jag2	jagged 2	-3.49	0.00046418
Jup	junction plakoglobin	-3.34	0.01174532
Lama3	laminin alpha 3	-53.20	4.6761E-23
Lamb3	laminin beta 3	-72.45	2.6791E-47
Lamc2	laminin gamma 2	-25.76	2.5374E-95
Lman1	lectin mannose-binding 1	-11.66	1.076E-58
Lman1l	lectin mannose-binding 1 like	-19.01	1.5223E-05
Ltb	lymphotoxin B	-11.19	0.02686554
Mapk13	mitogen-activated protein kinase 13	-2.88	0.00198238
Marcks	myristoylated alanine rich protein kinase C substrate	-18.09	0.00101276
Marveld2	MARVEL (membrane-associating) domain containing 2	-16.15	1.8456E-09
Marveld3	MARVEL (membrane-associating) domain containing 3	-28.10	1.1183E-07
Mlf1	myeloid leukemia factor 1	-6.31	0.02682574
Mmp13	matrix metallopeptidase 13	-9.91	0.00523828
Mras	muscle and microspikes RAS	-4.96	2.0199E-17
Msn	moesin	-2.35	1.579E-12
Myh14	myosin heavy polypeptide 14	-10.25	2.7546E-12
Nfatc2	nuclear factor of activated T cells cytoplasmic calcineurin dependent 2	-3.51	0.01019063
Ngef	neuronal guanine nucleotide exchange factor	-22.35	3.9983E-17
Nod2	nucleotide-binding oligomerization domain containing 2	-4.92	0.00901741
Nrp1	neuropilin 1	-2.53	0.0479032

Ntn4	netrin 4	-7.97	0.00513535
Nupr1	nuclear protein transcription regulator 1	-2.62	0.03313426
Pard6b	par-6 family cell polarity regulator beta	-2.62	0.01305986
Patj	PATJ crumbs cell polarity complex component	-2.64	0.00019806
Pdgfb	platelet derived growth factor B polypeptide	-2.73	0.01063051
Perp	PERP TP53 apoptosis effector	-87.69	1.451E-128
Pfn2	profilin 2	-5.99	0.00101979
Pla2g12a	phospholipase A2 group XIIA	-2.17	0.00202825
Plaat3	phospholipase A and acyltransferase 3	-20.28	1.5071E-29
Plxnb1	plexin B1	-4.02	1.6909E-06
Porcn	porcupine O-acyltransferase	-3.72	0.00753547
Ptprf	protein tyrosine phosphatase receptor type F	-2.08	1.4763E-05
Rapgef5	Rap guanine nucleotide exchange factor (GEF) 5	-29.37	4.5958E-07
Rassf5	Ras association (RalGDS/AF-6) domain family member 5	-2.50	0.02817663
Rnf43	ring finger protein 43	-48.92	5.0138E-08
Rps6ka6	ribosomal protein S6 kinase polypeptide 6	-26.99	1.1304E-06
Scin	scinderin	-30.55	1.1083E-15
Sema4d	sema domain immunoglobulin domain (Ig) transmembrane domain (TM) and short cytoplasmic domain (semaphorin) 4D	-2.76	0.00096864
Sema6a	sema domain transmembrane domain (TM) and cytoplasmic domain (semaphorin) 6A	-20.43	0.00308379
Serpine1	serine (or cysteine) peptidase inhibitor clade E member 1	-5.95	1.3956E-20
Sfn	stratifin	-34.36	1.4717E-24
Sgk2	serum/glucocorticoid regulated kinase 2	-5.08	0.01199091
Sgk3	serum/glucocorticoid regulated kinase 3	-12.32	4.2252E-06
St14	suppression of tumorigenicity 14 (colon carcinoma)	-28.49	4.406E-60
Steap3	STEAP family member 3	-19.53	9.0965E-06
Tjp2	tight junction protein 2	-3.33	6.2084E-06
Tjp3	tight junction protein 3	-70.55	4.7036E-12

Tnc	tenascin C	-23.33	1.0459E-05
Tnfaip3	tumor necrosis factor alpha-induced protein 3	-3.74	4.6215E-08
Tnfrsf9	tumor necrosis factor receptor superfamily member 9	-9.89	0.0005496
Wnt10a	wingless-type MMTV integration site family member 10A	-3.73	8.1493E-05
Wnt7a	wingless-type MMTV integration site family member 7A	-44.24	1.1828E-16
Wnt9a	wingless-type MMTV integration site family member 9A	-6.46	1.0139E-05

Supplementary Table S3. sgRNA oligos

	Sense	Anti-sense
Pair #1	ACGTTGCTGATACTGTCACC	GGTGACAGTATCAGCAACGT
Pair#2	GTATCAGCAACGTCAAGCAA	TTGCTTGACGTTGCTGATAC
Pair #3	GATCATGGGTTATGGACTGC	GCAGTCCATAACCCATGATC
Pair #4	TATCAGCAACGTCAAGCAAC	GTTGCTTGACGTTGCTGATA
Pair #5	GGAAGATTCTGAAGAAGAGA	TCTCTTCTTCAGAATCTTCC

Supplementary Table S4. Primers used for Real-Time qPCR (HyLabs, Israel and Sigma-Aldrich).

Gene products	FORWARD	REVERSE
HPRT	AGCTACTGTAATGATCAGTCAACG	AGAGGTCCTTTTCACCAGCA
IL-1α	GTTACAGTGAAAACGAAGACTAC	TGCAAGTCTCATGAAGTGAGC
IL-1β	TGCCACCTTTTGACAGTGATG	TGTGCTGCTGCGAGATTTGA
TGF-β	GGCTGAACCAAGGAGACGGA	GGTTCATGTCATGGATGGTGC
IL-6	CCACTTCACAAGTCGGAGGC	TTTCTGCAAGTGCATCATCGTT
GM-CSF	TGCCTGTCACGTTGAATGAAG	GAAATTGCCCCGTAGACCCT
M-CSF	GGCTCTTCAGCCACTAGCGA	GAGCCCAGCCATGTCGAAGA
G-CSF	AAGTCCCTGGAGCAAGTGAG	AGCTGGCTTAGGCACTGTGT
CXCL-1	AAACCGAAGTCATAGCCACAC	TCTCCGTTACTTGGGGACAC
CXCL-2	CCCAGACAGAAGTCATAGCCAC	TGGTTCTTCCGTTGAGGGAC
CXCL-3	CCAGACAGAAGTCATAGCCAC	CTTCATCATGGTGAGGGGCTT
CCL-2	AGGTCCCTGTCATGCTTCTG	TCTGGACCCATTCCTTCTTG
CCL-5	CCAATCTTGCAGTCGTGTTTGT	ACCCACTTCTTCTCTGGGTTG
PDGF-A	GGGACCTCCAGCGACTCTT	TGCAGGAATGGCTTCCTCAATAC
PDGF-B	CGAAAGAAGCCCATCTTCAA	CCTTGTCATGGGTGTGCTTA
VEGF-A	CTCCACCATGCCAAGTGGTC	GTCTCAATCGGACGGCAGTA
PD-L1	TCTCCTCGCCTGCAGATAGTT	ACAAGTCCTTTGGAGCCGTG
TSLP	GGCGACAGCATGGTTCTTC	ACAGTCCTCGATTTGCTCGAA
CXCR1	GCCTTTTGCTATCTTCCGCC	AGCAGACCAGCATAGTGAGC
CXCR2	GGGTCGTACTGCGTATCCTG	AGACAAGGACGACAGCGAAG

CXCR3	ATGGGGTCTCTGTCTGCTCT	TGAGGCGCTGATCGTAGTTG
CXCR4	TTACCCCGATAGCCTGTGGA	CAGGAGAGGATGACGATGCC
CCR5	ACTGCTGCCTAAACCCTGTC	ATGTTCTCCTGTGGATCGGG
CCR6	GGCAGTGTGGTTCATCTCCA	GTGGCTCACAGACATCACGA
CXCL5	CGGTTCCATCTCGCCATTCA	AAGCAAACACAACGCAGATC
CXCL9	GAGCAGTGTGGAGTTCGAGG	AGGCAGGTTTGATCTCCGTT
CXCL10	AGTGCTGCCGTCATTTTCTG	TCCCTATGGCCCTCATTCTCA
Bcl-2	CCTGTGGATGACTGAGTACCTG	AGCCAGGAGAAATCAAACAGA GG
BAX	CCAAGAAGCTGAGCGAGTGT	ACGTCAGCAATCATCCTCTGC
Granzyme-b	GGGGAGATCATCGGGGGACA	GCCCCCACATATCGCCTCAG
E-Cadherin	TCGCCACAGATGATGGTTCA	TTCGAGGTTCTGGGATGGGA
Vimentin	AGGCGAGGAGAGCAGGATTTC	CCATCTCTGGTCTCAACCGTC
β-Catenin	AATGAAGGCGTGGCAACATAC	GGCACCAATGTCCAGTCCAA
EGFR	GCAATGTTCCCATCGCTGCTGTC	CAGGTGTCTTTGCATGTGGC

Supplementary Table S5: List of antibodies

Target	Clone	Conjugation	Supplier	Cat. #
CD3	17A2	BG Violet 450	Biogems	05112-40-25
CD3	145-2C11	APC	eBioscience	17-0031-83
CD4	RM4-5	BG Violet 450	Biogems	06122-40-100
CD4	GK1.5	PE/cyanine7	eBioscience	25-0041-81
CD8a	53-6.7		eBioscience	14-0081-82
CD8a	53-6.7	PE/cyanine7	Biogems	10122-77-100
CD8a	53-6.7	APC/cyanine7	Biogems	10122-87-100
CD11b	M1/70	Pacific Blue	Biolegend	101224
CD11b	M1/70	PerCP/cyanine5.5	Biogems	03221-70-100
CD11b	M1/70	PE/cyanine7	Biogems	03221-77-100
CD11c	N4-18	APC/cyanine7	Biogems	03212-87-100
CD11c	N4-18	APC	Biolegend	117310
CD11c	N4-18	eFluor450	eBioscience	48-0114-82
CD11c	N4-18	PE	Biolegend	117308
NKp46	29A 1.4	PE/cyanine7	Biolegend	137618
MHC II	M5/114.15.2	PE/cyanine7	Biogems	86212-77-100
MHC II	M5/114.15.2	APC	Biogems	86212-80-100
MHC II	M5/114.15.2	PE	eBioscience	12-5321-81
Ly6c	HK1.4	APC/cyanine7	Biolegend	128026
Ly6c	HK1.4	PE cyanine7	eBioscience	25-5932-82

Ly6g	1A8	Alex Fluor649	Biolegend	127610
Ly6g	RB6-8C5	PE	Biogems	83122-60-100
F4/80	BM8		eBioscience	14-4801-82
F4/80	BM8	PE cyanine7	Biolegend	123114
F4/80	BM8	APC/cyanine7	Biolegend	123118
B220	RA3-6B2	PE	Biogems	07131-60-100
B7.1	16-10A1	APC	eBioscience	17-0801-81
B7.2	GL-1	Pacific Blue	Biolegend	105022
CD103	2E7	APC	Biogems	15112-80-25
CX3CR1	SA011F11	PE	Biolegend	149006
Neutrophil Elastase			Invitrogen	PA5-115648
CD64	X54-5/7.1	APC	Biolegend	139306
IL-1α	ALF161		Biolegend	503202
IL-1α	ALF161	PE	Biolegend	503203
Isotype Control		PE	Biolegend	400908
Armenian Hamster IgG		Alexa Fluor 647	Abcam	ab173004
