# Supplementary material for: Tumor Cell-Associated IL-1α Affects Breast Cancer Progression and Metastasis in Mice through Manipulation of the Tumor Immune Microenvironment
Source: Int J Mol Sci. 2024 Apr 2;25(7):3950. doi: 10.3390/ijms25073950 (PMC11011794; doi:10.3390/ijms25073950)
Supplement: Supplementary file 1 [file ijms-25-03950-s001.zip › Supplementary figures.pdf]

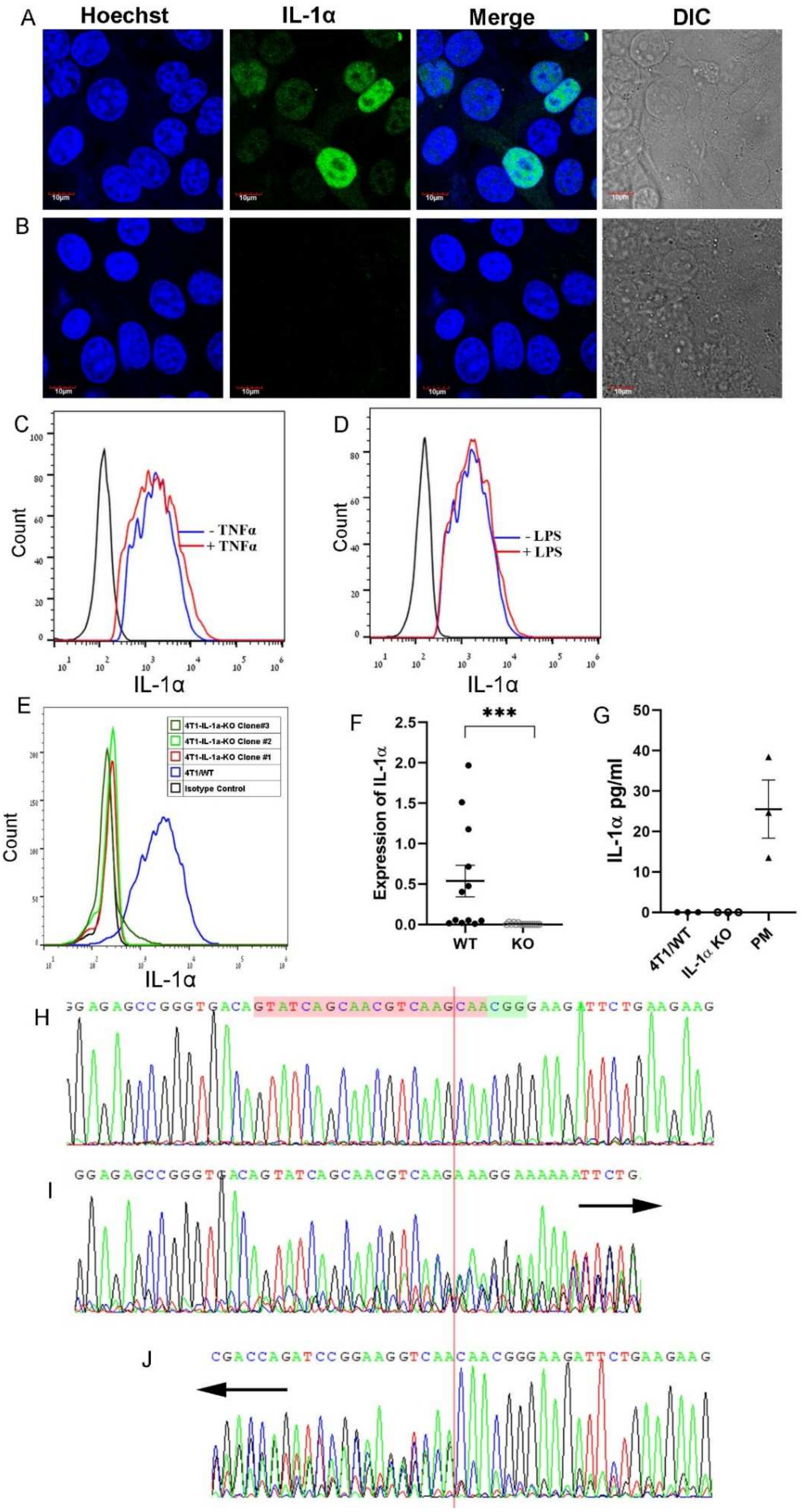

Supplementary figure S1. Endogenous expression of IL-1α in 4T1 cells and creation of 4T1 IL-1α KO cells Confocal microscope evaluation of 4T1/WT cells (A) and 4T1 IL-1α KO cells (B) stained with either PE-

conjugated anti-IL-1α-PE or isotype control antibodies. (C, D) 4T1/WT cells were treated with TNFα (50ng/ml) or LPS (100ng/ml) for 12 hours and production of IL-1α was analyzed by FC, using indicated antibodies. (E) Expression of IL-1α in Cas9 transfected IL-1$\alpha^{+/+}$ 4T1 cells (4T1/WT) and three distinct 4T1 IL-1α KO clones. (F) Expression of IL-1α in indicated cell lines was assessed by qPCR, normalized to HPRT1. The results are shown as average over all 3 KO clones ± SEM. Significant differences are indicated (unpaired 2-tailed t-test, ***$p<0.001$). (G) 4T1/WT, IL-1α KO clone 2 cells or murine peritoneal macrophages (PM) were seeded in flasks at 70% confluency, the supernatants were collected after 24 h and analyzed for IL-1α by ELISA. The results are shown as average ± SEM. (H-J) An example of genomic DNA sequencing results obtained from either 4T1/WT cells (H) or IL1α-KO clone 2 (I –forward direction, J – reversed direction). The region corresponding to exon 4 of IL-1α is shown. The sgRNA sequence targeting this region is highlighted in H (pink + green for PAM). The pink vertical line indicates the presumed double-strand break (DSB) site, created by CRISPR-Cas9.

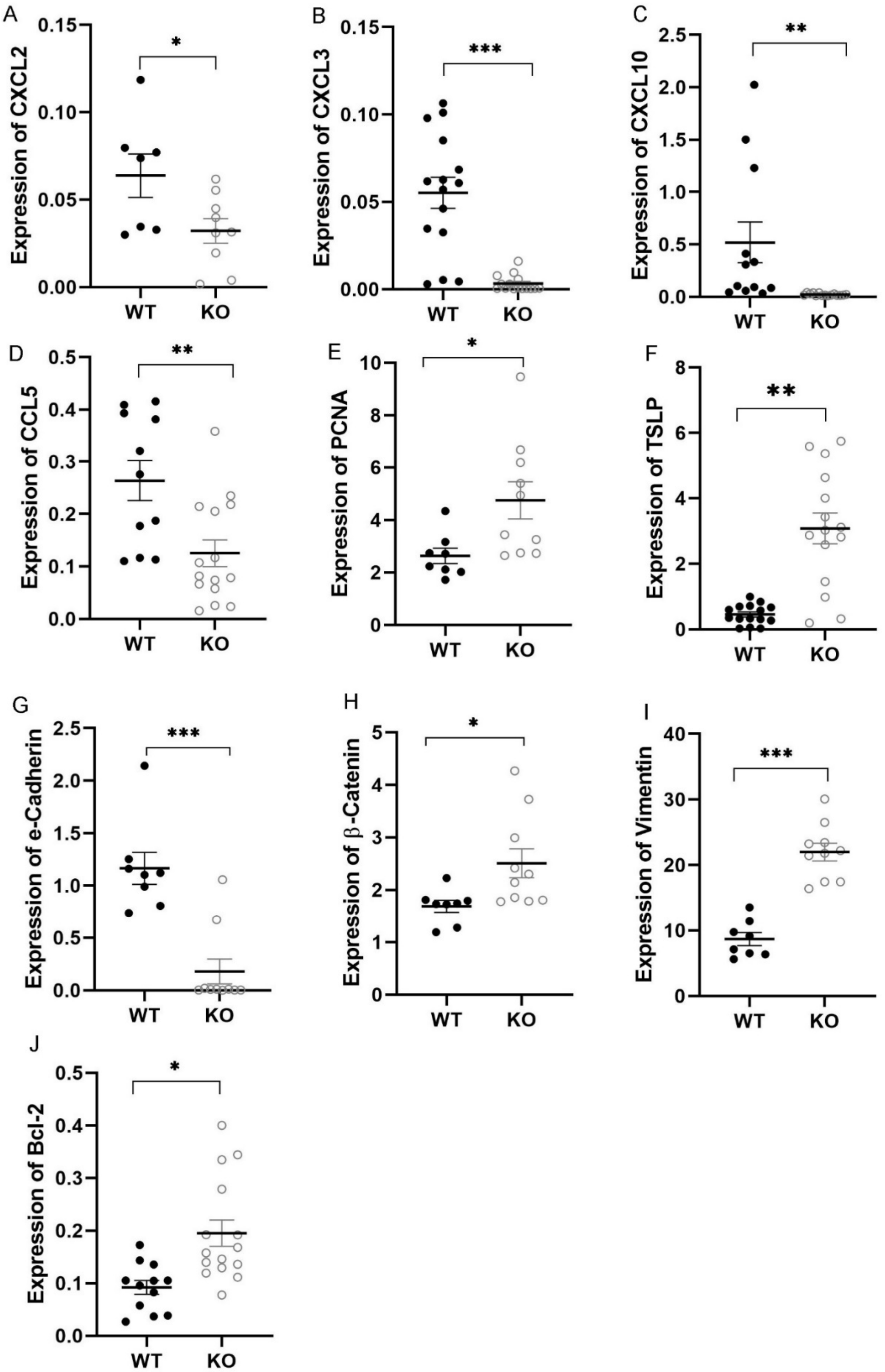

Supplementary figure S2. Effects of IL-1α knockout on gene expression in 4T1 cells. Expression of indicated cytokines in either 4T1/WT (WT) or 4T1 IL-1α KO (KO) was assessed using qPCR, normalized to HPRT1. The results are shown as average over all 3 KO clones ± SEM, experiments were performed on at least 2 separate cultures/lysates. Significant differences are indicated (unpaired 2-tailed t-test, *p<0.05, **p<0.01, ***p<0.001).

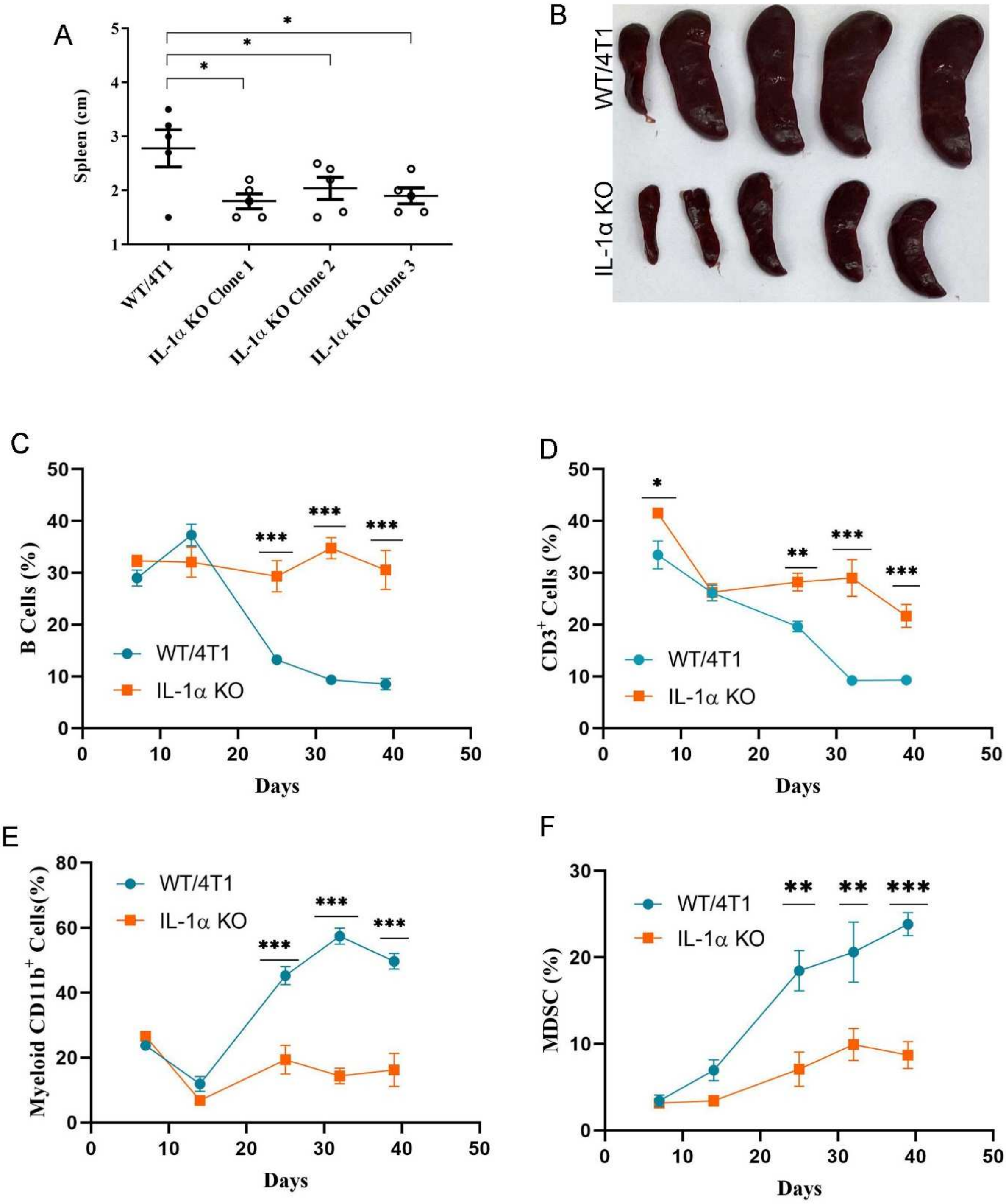

Supplementary figure S3. IL-1α knockout in 4T1 cells affects spleen alterations in tumor-bearing mice. 4T1/WT or 4T1 IL-1α KO clones were injected into BALB/c mice as described above - a representative experiment with 5 mice per group per time point (A) Weight of spleens harvested on day 30 post-injection. (B) Representative images of spleens harvested on day 30 post-injection. Single spleen cells were obtained and analyzed by FC. (C) Percentage of $B220^+$ cells in splenic tissue. (D) Percentage of $CD3^+$ cells in splenic tissue. (E) Percentage of $CD11b^+$ cells in splenic tissue. (F) Percentage of MDSC (defined as a combination of MHC-II$^-$CD11b$^+$Ly6G$^+$Ly6C^{low} and MHC-II$^-$CD11b$^+$Ly6G$^-$Ly6C^{high}) in splenic tissue (averaged over two experiments). The results are shown as average ± SEM. Significant differences are indicated (unpaired 2-tailed t-test, ***p<0.001).

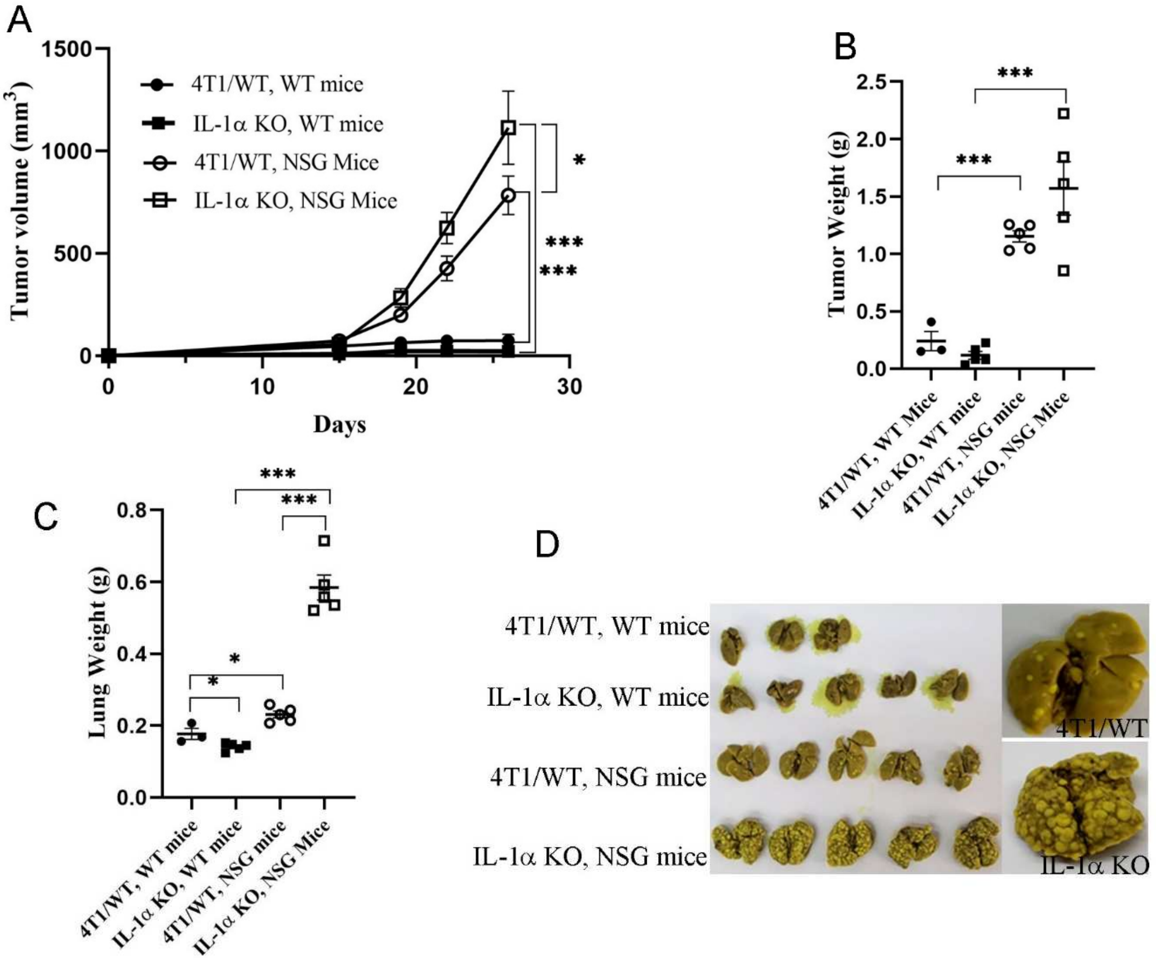

Supplementary figure S4. The functional immune system of the host is critical for inhibition of tumor development in mice engrafted with 4T1 IL-1*α* KO cells. 4T1 cells were injected as described above into either BALB/c (WT) or NSG mice – at least 4 mice per group. (A) The dynamics of tumor development following injection of indicated tumor lines into indicated hosts. Weights of tumors (B) and lungs (C) harvested on day 26 post injection. (F) Representative images of lungs harvested on day 26 post-injection. Each experiment included six mice per group, repeated twice. The results are shown as average ± SEM. Significant differences are indicated (2-way ANOVA (A) or unpaired t-test (B, C), *p<0.05, **p<0.01, ***p<0.001).
